# *JMLA* virtual projects continue to show impact of technologies in health sciences libraries

**DOI:** 10.5195/jmla.2025.2102

**Published:** 2025-01-14

**Authors:** Emily Hurst

**Affiliations:** 1 ehurst@hshsl.umaryland.edu, Virtual Projects Advisory Committee, Journal of the Medical Library Association, and University of Maryland Baltimore, Health Sciences and Human Services Library

## Abstract

Beginning in 2012, the Virtual Projects section of the *Journal of the Medical Library Association* has provided an opportunity for library leaders and technology experts to share with others how new technologies are being adopted by health sciences libraries. From educational purposes to online tools that enhance library services or access to resources, the Virtual Projects section brings technology use examples to the forefront. The new publication issue for future Virtual Projects sections will be January and the call for submissions and Virtual Projects deadline will now take place in June and July.

The Virtual Projects Section continues to evolve, enlighten, and offer a forum for health sciences libraries to share their ongoing work with technology-focused projects. This year the *JMLA* Virtual Projects Committee was delighted by the number of project abstracts received. Since 2020, those submitting content proposals for the Medical Library Association (MLA) conference may opt in to have their abstract reviewed by the *JMLA* Virtual Projects Committee for consideration in the Virtual Projects Section. This opportunity has opened the door, allowing even more projects to be considered.

This year the Virtual Projects Committee is pleased to share eight projects that demonstrate the wide depth and breadth of technology deployment in health sciences libraries. A number of projects this year demonstrate early adoption of artificial intelligence (AI). From the use of AI to facilitate event planning to AI assistance in collection development as well as AI use in library research guide creation, AI is transforming nearly every aspect of health sciences librarianship. Also of note, this year several projects demonstrate the robust use of technology to enhance teaching and training including both in-person sessions and online modules. Unique ways to support the evolution and training of bioinformatics are also showcased in one project. Another project provides perspectives on the use of free and paid tools to quickly create and maintain clinical bibliographies and technology solutions to enhance repositories through the creation of digital object identifiers (DOIs) are highlighted in another.

Material for this year's column was selected in 2024 with the assistance of the *JMLA* Virtual Projects Advisory Committee: Christine Andresen; Emily J. Hurst section editor; Michelle Kraft, AHIP, section coeditor; J. Dale Prince, AHIP; Tariq Rahaman; and Brian Zelip. Selected projects were edited by Emily J. Hurst.

